# A vision-based multi-camera fusion and CAD projection framework for risk-point identification in metro stations

**DOI:** 10.1038/s41598-026-50932-1

**Published:** 2026-05-07

**Authors:** Yichao Pu, Qianqi Fan, Shengyu Zhang, Qi Zhang, Jianyong Zuo

**Affiliations:** 1 College of Electronic and Information Engineering, Tongji University, Shanghai, 201804 China; 2 Institute of Rail Transit, Tongji University, Shanghai, 201804 China; 3 Shanghai Shentong Metro Group, Shanghai, 200333 China; 4 Wan Lian Yi Da Group, Beijing, 100015 China

**Keywords:** Urban rail transit, Crowd risk detection, Multi-camera fusion, Engineering, Mathematics and computing

## Abstract

Metro systems face challenges in managing localized congestion and ensuring passenger safety, particularly under high-density passenger flows and unexpected disruption scenarios. Conventional crowd detection methods often fail due to severe occlusion, insufficient coverage, and excessive latency, making them unreliable for providing operators with accurate risk identification and control support. To address this fundamental problem, this paper proposes a vision-driven framework for risk-point identification tailored to the complex spatial structure of metro stations. The framework adopts a world-coordinate-first multi-camera fusion strategy combined with CAD (Computer-Aided Design) floor-plan projection to achieve station-wide coverage and spatial interpretability; it introduces a density-speed dual-factor risk index to overcome the limitations of single-density indicators in capturing dynamic anomalies; and it incorporates a baseline-increment mechanism to effectively distinguish recurrent congestion from emerging anomalies, supporting fine-grained risk management. Empirical studies at two representative transfer hubs in Shanghai—Station A and Station B—demonstrate that the framework consistently achieves recall rates above 0.9 and 100% coverage of risk points in both routine and disruption scenarios, while sustaining real-time performance at 28 FPS. These results confirm that the proposed framework effectively bridges the gap between macroscopic passenger flow forecasting and microscopic risk sensing, offering a promising technical foundation for pilot-stage operational safety management and fine-grained risk sensing.

## Introduction

Urban rail transit systems are indispensable for sustaining the mobility, safety, and resilience of modern cities. Yet, unexpected disruptions, sudden passenger surges, and localized congestion events continue to threaten operational stability and passenger safety. A fundamental question for both researchers and practitioners is how to achieve reliable, fine-grained risk identification inside complex metro stations, under both routine and disruption scenarios. This question goes beyond traditional demand forecasting, as it requires not only predicting passenger volumes but also detecting where and when risks emerge within the intricate spatial structures of transfer corridors, escalators, and ticket gates.

Over the past decade, researchers have explored multiple approaches to improve passenger flow monitoring and crowd risk detection inside metro stations. One line of work relies on sensing-based macroscopic flow estimation. For example, Wi-Fi probe data have been widely used to reconstruct large-scale origin–destination flows and to support short-term demand forecasting, especially when combined with ticketing records and external data using graph neural networks^1–4^. These approaches provide good coverage at the network level, but their effectiveness is constrained by device carrying heterogeneity, MAC address randomization, and signal obstruction. As a result, they often introduce systematic bias and lack the spatial granularity needed to identify localized risks within transfer corridors, bottlenecks, or gates.

A second line of work focuses on computer vision-based crowd analysis, which offers the fine spatial resolution required at the station level. Transformer-based detectors, such as MPDNet^5^, have improved robustness to occlusion and perspective distortion in crowded metro environments, while density-estimation networks with self-attention have enhanced counting accuracy in heavily congested scenes^6,7^. Real-time object detection models like YOLOv7, YOLOv8^8^, and RT-DETR balance speed and accuracy, enabling near real-time tracking of passenger flows^9,10^. In parallel, optical flow models such as RAFT and FlowFormer + + can reconstruct dense motion fields and capture sudden crowding, backflows, or surges^11,12^. However, these vision-only methods still face difficulties in extreme crowding or low-light conditions, and their performance often degrades when scaled up to large station environments, where both latency and spatial coverage become critical bottlenecks.

To overcome these limitations, a third line of work emphasizes multimodal fusion and collaborative architectures. Studies combining UWB, Wi-Fi, and vision data have shown reduced positioning errors and improved continuity in complex indoor environments^13,14^. Edge-cloud collaborative pipelines distribute lightweight detection tasks to edge devices while reserving global inference for cloud servers, which enables near real-time alerts for large-scale crowd gathering^15^. Furthermore, advances in multi-camera video stitching now make it possible to generate panoramic views across concourses and platforms, reducing ID switches across cameras and supporting more consistent density estimation^16–18^. Yet, these pipelines are still hindered by synchronization issues, photometric inconsistencies, and seam artifacts, which restrict their practical deployment for continuous, station-wide risk monitoring.

Despite these advances across sensing, vision, and fusion-based approaches, critical challenges remain for operational deployment in metro environments. Metro stations are characterized by extremely high passenger densities, sudden disruption scenarios, and complex spatial bottlenecks such as escalators and transfer corridors. Under these conditions, even advanced single-camera methods often struggle with missed or duplicated detections due to extreme occlusion. While multi-camera fusion expands spatial coverage, it frequently faces issues of temporal misalignment, dynamic object blending, and computational latency, hindering seamless real-time operation. More importantly, most existing approaches stop at producing visual outputs without mapping results to the station’s CAD floor plans. This limits their operational utility, as metro operators require spatially precise, actionable insights that reflect not only crowd density but also abnormal movement dynamics. Consequently, the challenge of achieving reliable, real-time, and operationally deployable risk-point identification tailored to high-density and disruption-prone metro environments remains a pressing issue.

To address the aforementioned research gaps, this study develops a unified, computer vision-driven framework for dynamic and spatially explicit risk point detection inside metro stations. The main contributions are:

1. Architectural Novelty: World-Coordinate-First Fusion and CAD Mapping. Rather than modifying base vision models, we design a world-coordinate-first multi-camera fusion pipeline that projects heterogeneous pixel-level detections onto a unified ground plane and aligns them with station CAD floor plans via robust homography. This bypasses traditional image-domain stitching, eliminating seam artifacts and ensuring physically interpretable, station-wide coverage.
2. Mechanism Innovation: Point-Flow Synergy and Risk Quantification. We introduce a point-flow collaborative mechanism that anchors discrete SORT (Simple Online and Realtime Tracking) trajectories within continuous PWC-Net optical flow fields, providing inertial compensation that reduces ID switches by 81.2% under extreme occlusion. Coupled with a density-speed dual-factor risk index and a baseline-increment detection mechanism, the framework dynamically decouples recurrent peak-hour congestion from disruption-induced anomalies.
3. Pilot-Scale Feasibility Validation: The system is validated in two major Shanghai transfer hubs under routine and disruption scenarios, achieving > 90% recall, 100% historical risk-point coverage, and sustained real-time performance (28 FPS). These results demonstrate the framework’s technical feasibility and potential for operational deployment, though broader validation across diverse network topologies is required for full-scale rollout.

This integrated pipeline is empirically validated in two representative Shanghai metro stations under routine and disruption scenarios, demonstrating its ability to bridge macroscopic forecasting with microscopic, real-time risk sensing and to support proactive passenger flow management.

## Problem statement and objectives

**Definition of Metro Station Risk. ** In metro station passenger flow management, risk refers to unsafe states formed by passenger crowds in specific spatial units, characterized by potential hazards caused by high density (spatial congestion) and abnormal speed (restricted movement or sudden surges). Its core identifying features are:

1. Excessive density: The number of passengers per unit area exceeds safety thresholds. For example, when density > 6 persons/m^2^, physical contact between individuals is unavoidable, and walking space is severely limited; when density ranges from 3 to 6 persons/m^2^, contact can be avoided but standing space remains tight, causing discomfort with prolonged stays.
2. Abnormal speed: The average walking speed drops significantly (e.g., < 0.3 m/s, indicating near-static crowd conditions) or abnormal movement behaviors occur, such as reverse walking, cross-traffic aggregation, or sudden stops—all of which increase the likelihood of crowd conflicts or stampedes.

**Quantitative Calculation of Risk.** To comprehensively integrate density and speed factors for objective risk assessment, this study proposes a risk index formula to quantify risk levels at spatial–temporal points:

1 $$R(X, t)=\alpha \cdot D(X, t) +\beta \cdot V(X, t),$$

where $R(X, t)$ represents the risk index at spatial coordinate $X$ and time $t$ (higher values indicate higher risk); $D(X, t)$ denotes passenger flow density at $(X, t)$(units: persons/m^2^), derived from the constructed crowd density heatmap; $V(X, t)$ is the average passenger speed at $(X, t)$ (units: m/s), estimated by the PWC-Net optical flow model in next section; following Fruin’s^19^ empirical thresholds for pedestrian level of service, we set $\alpha$=0.7, $\beta$=0.3 in this study to reflect the relative contribution of density and speed to risk. A sensitivity analysis confirms that the framework operates within a robust performance plateau around these parameters (Table 4). Given that the core contribution of this work lies in the world-coordinate fusion pipeline and CAD-interpretable spatial mapping, the risk index is treated as a modular component. Advanced formulations, such as non-linear density-speed coupling or data-driven adaptive weighting, will be systematically explored in future work to further enhance context-aware anomaly detection.

A region is identified as a “risk point” when $R(X, t)$ exceeds a predefined threshold (calibrated based on historical safety incidents and operational standards. To address the aforementioned research gaps and support the above-defined crowd risk detection, this study develops a unified, computer vision-driven framework for dynamic and spatially explicit risk point detection inside metro stations. The objectives are threefold:

1. Single-camera microscopic sensing to perform robust passenger detection and tracking under high density by combining state-of-the-art real-time detectors with lightweight optical flow estimation for reconstructing continuous passenger vector fields, laying the foundation for accurate calculation of $D(X, t)$ and $V(X, t)$.
2. Multi-camera fusion and panoramic view construction to spatially stitch density maps from fixed and omnidirectional cameras in real time, enabling continuous, station-wide visualization of crowd distribution and movement, and solving the problem of fragmented data affecting risk assessment accuracy.
3. CAD-based spatial risk mapping to project the fused density and motion information onto the station CAD floor plan, generating a dynamic list of risk hotspots that can be directly used by station operators for early warning, operational adjustment, and emergency response.

## Methodology

The proposed framework is designed to close the research gaps identified in previous section by integrating three key stages: (i) robust single-camera passenger detection and tracking, (ii) multi-camera fusion and panoramic view construction, and (iii) CAD-based spatial risk mapping. Together, these components form a unified, real-time pipeline that bridges macroscopic flow forecasting with microscopic risk sensing.

In this research, FCOS (anchor-free detection), SORT (Kalman-filter-based tracking), and PWC-Net (dense optical flow) are utilized as computational backbones selected for their proven efficiency and robustness. Our methodological contributions lie in (i) orchestrating them through a world-coordinate-first fusion strategy that projects heterogeneous video feeds onto a unified, CAD-registered ground plane, thereby eliminating image-domain stitching distortions; (ii) developing a point-flow synergy mechanism that leverages continuous optical flow fields for trajectory inertial compensation, significantly mitigating occlusion-induced tracking failures; and (iii) formulating a density-speed dual-factor risk index integrated with a baseline-increment mechanism that translates raw perception outputs into spatially explicit, operator-actionable risk scores. This system-level integration transforms off-the-shelf vision modules into a deployment-ready risk identification framework tailored to the high-density, low-fault-tolerance constraints of metro environments.

**Fig. 1:**
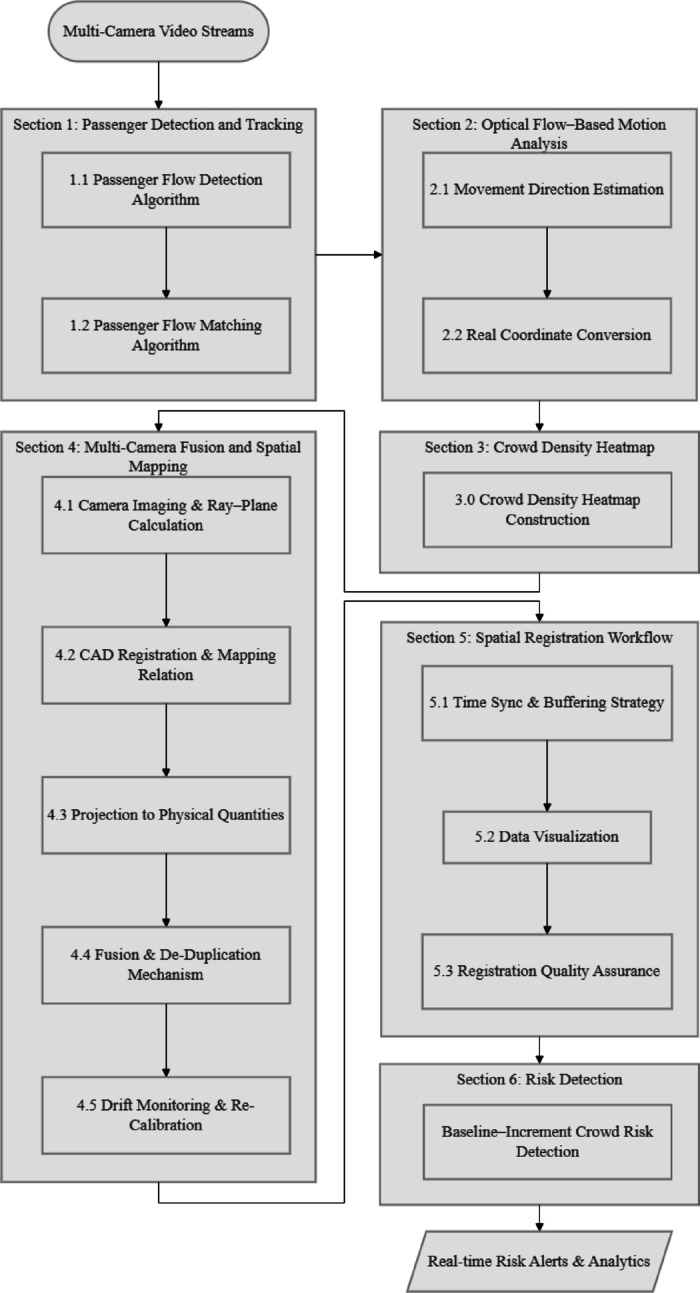
Overall system pipeline of the proposed vision-driven risk-point identification framework.

Figure 1 illustrates the end-to-end workflow of the proposed framework. The system processes multi-camera video streams through six sequential stages, each corresponding to a major section of this methodology description.

**Passenger Detection and Tracking.** To balance real-time performance with detection robustness under high-density conditions, we employ a lightweight FCOS variant as the perception backbone. Instead of the standard ResNet-50, a simplified 3-stage convolutional backbone coupled with a Feature Pyramid Network (FPN) is adopted, reducing computational overhead by about 60% while maintaining an mAP of 87.4% on our metro-specific dataset. The detailed architecture specifications and loss formulation (Eqs. 8 and 9) are provided in Appendix 1.

For multi-object tracking, we integrate the SORT framework to provide a lightweight trajectory baseline. SORT utilizes a Kalman filter for state prediction and Hungarian algorithm for IoU-based association, ensuring identity consistency across frames with minimal latency. Crucially, in our framework, SORT does not operate in isolation; its discrete trajectory outputs serve as anchor points for the subsequent point-flow synergy mechanism, where continuous optical flow fields compensate for occlusion-induced fragmentation. The standard Kalman state formulation (Eq. 10) is omitted here for brevity and included in Appendix 1.

**Optical Flow-Based Motion Analysis.** To capture microscopic motion trends in occlusion-prone regions, we integrate PWC-Net to generate dense, sub-pixel optical flow fields. Unlike discrete detection boxes, PWC-Net’s continuous motion estimation remains robust when physical overlap obscures individual features—a frequent occurrence in metro peak-hour scenarios. The network’s pyramid feature extraction and warping mechanisms enable real-time inference (about 35 FPS on Sintel benchmark), providing the velocity field input required for our risk quantification module. Training details and loss functions (Eqs. 11 and 12) are documented in Appendix 2.

**Crowd Density Heatmap Construction.** Accurate spatial mapping of passenger distribution is foundational for risk-point identification. In high-density metro environments where severe occlusion limits traditional counting methods, we employ an adaptive kernel density estimation approach to construct a continuous crowd probability heatmap. This method dynamically adjusts the spatial influence range of each detected passenger based on local neighborhood density, concentrating resolution in congested bottlenecks while stabilizing estimates in sparse areas. The resulting density field is subsequently coupled with pixel-level optical flow magnitudes to generate a speed-density aggregation map, capturing both spatial congestion and rapid movement anomalies. The complete mathematical formulation (Eqs. 13 and 14), including the adaptive Gaussian kernel strategy, parameter constraints, and feature coupling logic, is provided in Appendix 3. This mapped density-velocity field serves as the primary spatial input for the dual-factor risk index and subsequent world-coordinate fusion pipeline.

**Multi-Camera Fusion and Spatial Mapping.** Building upon the lightweight perception backbones described above, the core innovation of our framework resides in the world-coordinate-first fusion strategy and risk-quantification mechanisms detailed below.

To achieve global spatial visualization of high-risk points inside metro stations, this study proposes a "world-coordinate-first" strategy. Instead of performing image-domain stitching as a preprocessing step, pixel-level representations from each camera are directly mapped to the ground plane, and multi-camera fusion and de-duplication are then carried out in the world coordinate domain. This approach avoids projection distortions caused by invalid planar assumptions and ensures that the output results are physically interpretable.

Given the heterogeneous camera setup in station concourses—typically comprising peripheral static cameras and a centrally mounted spherical camera—direct image-domain stitching often results in data discontinuities due to differing coordinate systems and distortion characteristics (e.g., radial distortion in spherical views). Therefore, a hierarchical fusion strategy is adopted: peripheral cameras are stitched first, then fused with the spherical projection in the world-coordinate domain to ensure a coherent passenger flow map.

The fusion pipeline is structured around seven sequential stages: (i) camera imaging and ray-plane intersection calculation; (ii) CAD registration via homography transformation; (iii) projection of density and velocity fields to ground-level physical quantities; (iv) confidence-weighted multi-camera fusion coupled with occupancy-based de-duplication; (v) drift monitoring and lightweight re-calibration for long-term stability; (vi) CAD-based spatial registration and projection workflow for operational visualization; and (vii) time synchronization and buffering for multi-stream temporal alignment. The following subsections detail each stage with corresponding mathematical formulations and engineering protocols.

***Camera imaging model and ray-plane intersection calculation.*** To map pixel-level observations to the station’s physical floor plan, each camera is first calibrated to obtain intrinsic and extrinsic parameters. Conventional cameras follow a standard pinhole projection model, while spherical cameras are modeled using a polynomial equidistant projection to correct radial distortion. After distortion correction, each pixel coordinate is converted into a normalized 3D direction vector originating from the calibrated optical center. The geometric intersection between this viewing ray and the approximated concourse floor plane yields the corresponding 3D ground coordinate. This projection step ensures that all subsequent multi-camera fusion and risk mapping operations are performed in a unified, physically interpretable world coordinate system. The complete mathematical formulations (Eq. 15–19) for camera imaging models, distortion correction, and ray-plane intersection derivation are provided in Appendix 4.

***CAD Registration and Planar Mapping Relationship.*** To represent passenger flow dynamics on a unified metro station concourse CAD plane, it is necessary to convert 3D floor points $(X,Y)$ into pixel coordinates $({u}_{cad}, {v}_{cad})$ on the CAD plane. This mapping is achieved using a homography matrix ${H}_{cad}$, as shown below:

2 $$s\left[\begin{array}{c}{u}_{cad}\\ {v}_{cad}\\ 1\end{array}\right]={H}_{cad}\left[\begin{array}{c}X\\ Y\\ 1\end{array}\right],$$

where $({u}_{cad}, {v}_{cad})$ denotes the pixel coordinates on the CAD plan, $(X,Y)$ represents the floor plane coordinates, and $s$ is the scaling factor. The homography matrix ${H}_{cad}$ is obtained via registration using four or more control points. These control points are usually selected from column bases, turnstile corners, or floor tile intersections to ensure the stability of spatial alignment.

***Projection to Ground-Level Physical Quantities.*** To ensure that risk assessment operates on physically interpretable metrics, pixel-level density heatmaps and optical flow fields must be projected onto the unified ground plane. Ground-level density is reconstructed via a conservation-preserving interpolation kernel that aggregates pixel contributions within each spatial grid cell. Concurrently, ground velocity vectors are derived by applying the inverse Jacobian of the camera projection function to the pixel optical flow, scaled by the inter-frame time interval. This geometric conversion ensures that all subsequent multi-camera fusion and risk-index calculations are performed in real-world units (persons/m^2^ and m/s). The complete mathematical formulations (Eq. 20–23) for density mapping, Jacobian derivation, and velocity conversion are provided in Appendix 5.

***Multi-Camera Fusion and De-Duplication Mechanism.*** Multiple cameras may cover the same area; therefore, a weighted fusion strategy needs to be designed. For each ground point $X$, the confidence weight is calculated as:

3 $$\widetilde{{{{w}}_{{{i}}} }}{{(X) = (\alpha cos}}\emptyset _{{{i}}} {{ + \beta }}\frac{{{1}}}{{{{d}}_{{{i}}} }}{{ + \gamma (1 - v}}_{{{i}}} {{) + \delta }}\frac{{{1}}}{{{{\sigma }}_{{{i}}}^{{{2}}} }}{{)M}}_{{{i}}} {{(X),}}$$

where. ${{\emptyset }}_{i}$ is the incident angle, ${d}_{i}$ is the distance from the camera to the point, ${v}_{i}$ denotes the vignetting coefficient, ${\sigma}_{i}^{2}$ is the uncertainty variance, and ${M}_{i}(X)$ represents the visibility mask. The coefficients, $\alpha , \beta , \gamma , \delta$, control the relative weights of each factor.

The density after weighted fusion is:

4 $${{\hat{D}(X,~t) = }}\frac{{\mathop \sum \nolimits_{{{i}}} \widetilde{{{{w}}_{{{i}}} }}{{(X)D}}_{{{i}}} {{(X,~t)}}}}{{\mathop \sum \nolimits_{{{i}}} \widetilde{{{{w}}_{{{i}}} }}{{(X) + \varepsilon }}}}{{,}}$$

where ε is a constant introduced to prevent division by zero. For velocity fusion, robust optimization is employed:

5 $${v}^{*}(X, t)=arg min \sum_{i}\rho (\Vert v-{v}_{i}(X, t)\Vert )\widetilde{{w}_{i}}(X),$$

where, ρ · is a robust kernel function. To avoid duplicate counting, an occupancy probability is introduced:

6 $${P}_{OCC}(X, t)=1-{\prod}_{i}(1-{p}_{i}(X, t)),$$

where ${p}_{i}(X, t)=1-{e}^{-\widehat{{D}_{i}}(X, t){A}_{cell}}$ is the occupancy probability from a single camera. In this context, $\widehat{{D}_{i}}$ represents the local density estimation, and ${A}_{cell}$ denotes the grid cell area.

***Drift Monitoring and Lightweight Re-Calibration Mechanism.*** During long-term operation, cameras may experience slight deviations due to vibrations or environmental factors. To ensure mapping accuracy, this method incorporates online drift monitoring. A set of fixed anchor points $\{{X}_{k}\}$ is selected, and the reprojection residual of these points on the image plane is computed as:

7 $$e_{{i,k}} = {||}p_{i} (X_{k} ) - {\mathbf{u}}_{{i,k}}^{{obs}} {||},$$

where π_i_(X_k_) is the projection of the anchor point onto the $i$-th camera, and ${\mathbf{u}}_{i,k}^{obs}$ represents the observed coordinates. When ${max}_{k}{e}_{i,k}>\tau$ and this condition persists for a certain number of $T$ frames, lightweight re-calibration is triggered—only the yaw angle and minor translational offsets are optimized to avoid significant system jitter. The updated Look-Up Table (LUT) is partially replaced to ensure stability.

**Fig. 2:**
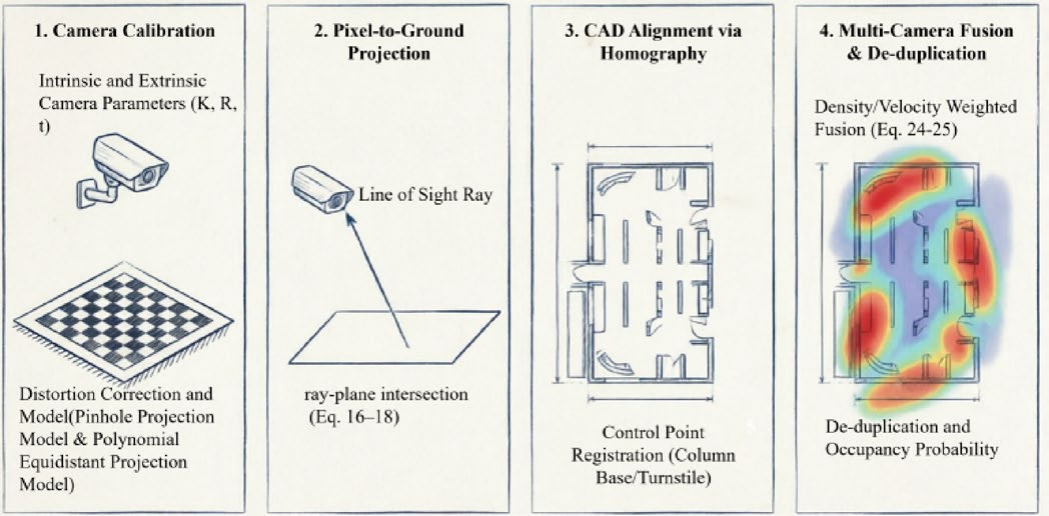
CAD-based spatial registration and projection workflow.

**CAD-Based Spatial Registration and Projection Workflow.** Figure 2 illustrates the four-step spatial registration workflow that bridges raw video data with the station’s architectural layout.

**Fig. 3:**
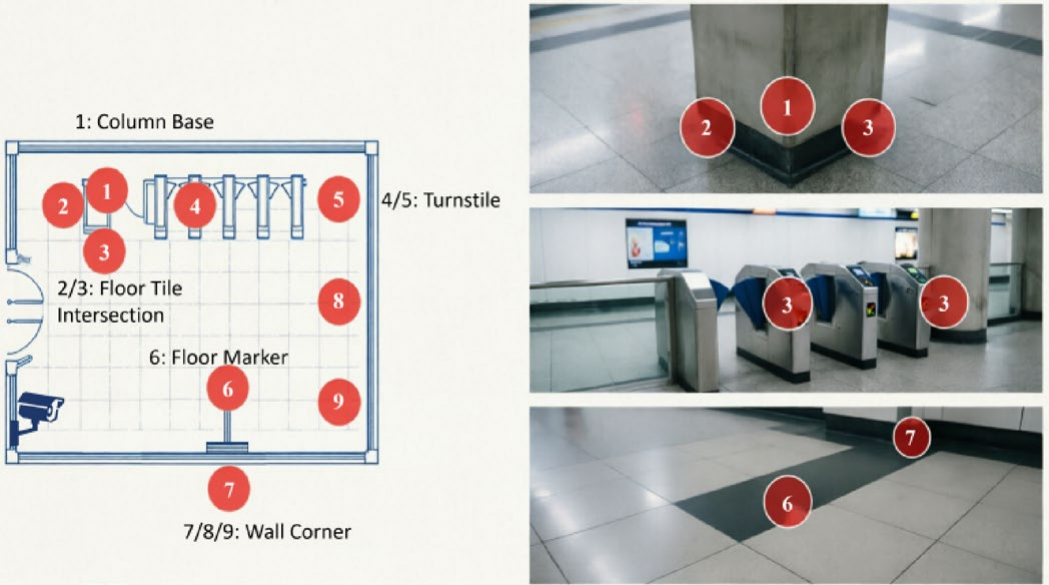
Example of control point selection for CAD registration.

First, intrinsic and extrinsic camera parameters are obtained via calibration (Eq. 15), distinguishing between conventional pinhole and spherical projection models. Second, pixel coordinates are projected to physical ground coordinates using ray-plane intersection (Eq. 17–19), converting image observations into real-world meters. Third, these ground coordinates are aligned with the station CAD floor plan via a coordinate transformation matrix (Eq. 2), a step critical for ensuring spatial interpretability. In reference to existing studies, Marendić et al.^20^ employed a configuration of six reference points (four edge points plus two center points) in UAV-based structural displacement monitoring to minimize homography transformation errors, while Alizadeh Naeini et al.^21^ utilized at least four wall intersections as control points for registering 3D point clouds to 2D CAD models. Targeting the station scenario, this study selects at least seven control points from stable structural features (e.g., column bases, gate terminals, and floor markers), as illustrated in Fig. 3, to ensure robust alignment and maintain residuals below 1.5 CAD pixels. Finally, within the unified CAD coordinate system, multi-camera observation data are fused using confidence-weighted averaging (Eqs. 3 and 4) to eliminate duplicate counting and mitigate occlusion errors.

From a deployment perspective, this registration process requires approximately 2–3 h of on-site work per station, primarily for control point annotation and homography validation. Following calibration, the system demonstrates stable continuous operation within the tested environments, with long-term field stability subject to further multi-site evaluation.

***Time Synchronization and Buffering Strategy.*** Differences may exist in the frame rates and timestamps of multiple cameras. To achieve accurate fusion at the same moment, time synchronization of video frames is required. Assume the target time is $t$; for each camera $i$, the nearest frame timestamp ${t}_{i}$ is selected. If $\left|t-{t}_{i}\right|<\Delta {t}_{max}$, this frame is used; otherwise, linear interpolation is applied to generate a virtual frame. This strategy ensures consistency of results across cameras under the same time axis, thereby preventing positional misalignment of high-speed moving crowds in overlapping regions. For real-time monitoring, a circular buffer with 3-frame depth is maintained per camera to accommodate network jitter while limiting end-to-end latency to < 100 ms. If synchronization fails beyond the 33 ms threshold, the system gracefully degrades to single-camera mode for affected regions, ensuring continuous risk detection without system-wide interruption.

***Data Visualization.*** Although data fusion is completed in the world coordinate domain, intuitive visualization results are still required in engineering applications to support decision-making by managers. To this end, an independent image-domain stitching branch is designed. This branch does not affect statistical metrics and is solely used for human visual inspection. Feature registration and multi-band fusion are applied between static cameras to generate a panoramic edge view; spherical cameras undergo cylindrical projection before being overlaid onto the stitched image. Finally, the entire result is uniformly mapped onto the CAD plane background. This approach not only provides accurate numerical results but also outputs a continuous and natural panoramic image.

**Fig. 4:**
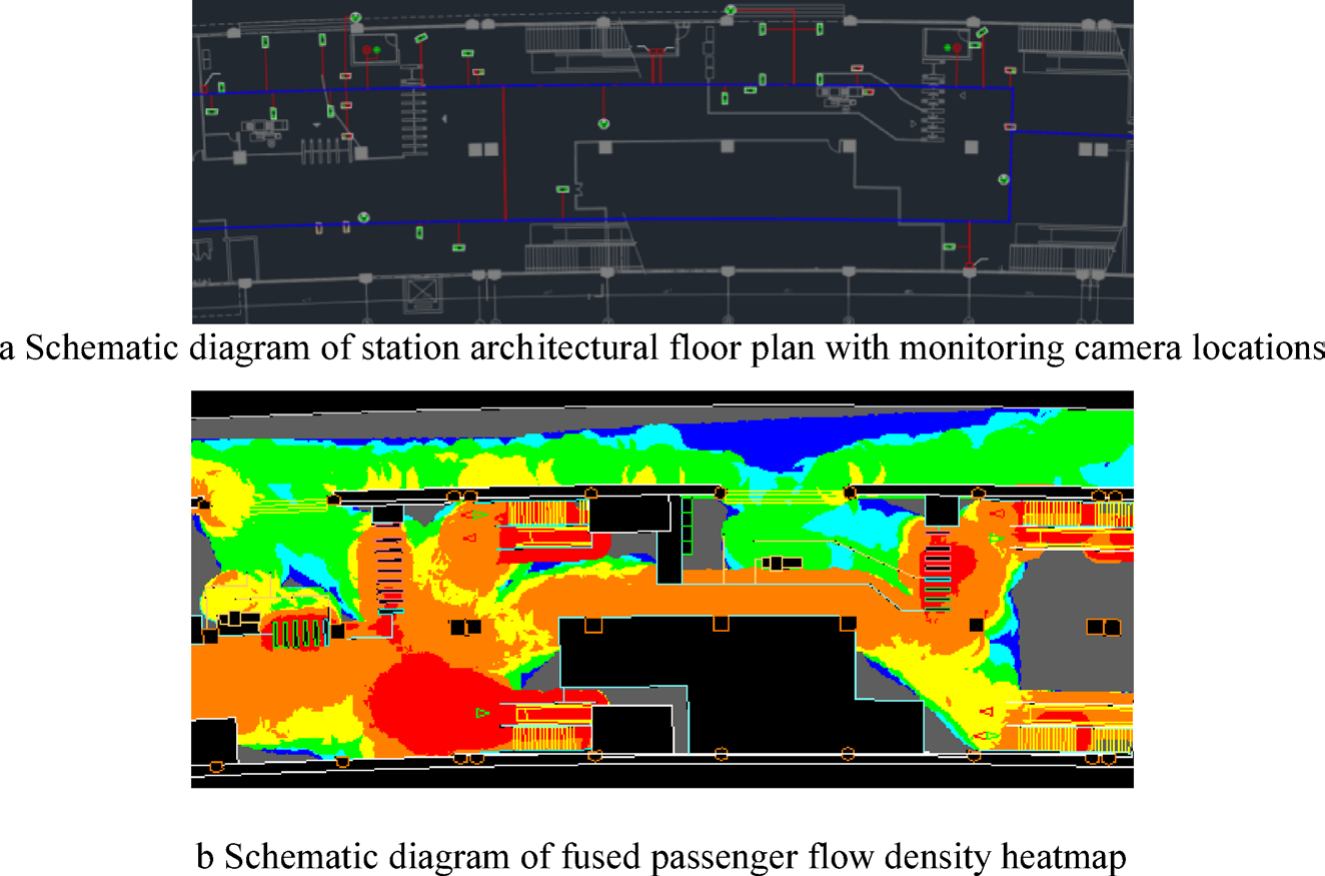
Fused station-wide crowd density heatmap overlaid on floor plan.

The resulting "full-station aggregation heatmap" (Fig. 4) accurately reflects risk level, overlaid on the floor plan to visualize dynamics across platforms, stairs, and escalators. This unified, time-series data supports subsequent risk scoring modeling by exposing structural bottlenecks and abnormal aggregation points during peak hours.

***Registration Quality Assurance.*** To ensure the reliability of CAD alignment, we implement a two-stage validation process. First, we compute the root-mean-square (RMS) registration residual across all control points; if the residual exceeds 1.5 CAD pixels, the homography estimation is repeated with additional control points. Second, we perform visual inspection by overlaying detected passenger trajectories onto the CAD floor plan and verifying that trajectories align with physical walkways and do not penetrate walls or obstacles. This quality assurance step is critical for maintaining spatial interpretability of the risk maps, as misalignment could lead to incorrect identification of risk locations (e.g., placing a risk point in a wall rather than a corridor).

**Baseline-Increment Crowd Risk Detection.** Static density and motion fields alone cannot fully capture the dynamic nature of passenger flow risks. To enhance adaptability, this study introduces a baseline-increment mechanism that integrates historical patterns with real-time anomalies.

1. Baseline Construction:
  Historical surveillance data are batch-processed to establish stable spatial distributions of passenger density and motion flow under normal operating conditions. These baseline maps characterize recurrent congestion zones (e.g., near transfer corridors, escalators, and ticket gates) and serve as reference distributions.
2. Incremental Anomaly Detection:
  Real-time passenger trajectories (from SORT) and motion vectors (from PWC-Net) are continuously compared against baseline maps. Deviations beyond statistically defined thresholds—such as abnormal density surges, unexpected counterflows, or sudden clustering in low-risk zones—are flagged as increments. These increments indicate emerging risks not present in the historical baseline, such as temporary bottlenecks or cascading congestion caused by service disruptions.

**Operational Constraints and Mitigation Strategies.** While the proposed framework demonstrates technical feasibility under pilot conditions, real-world metro deployment requires explicit acknowledgment of operational constraints and predefined mitigation protocols. Four primary engineering constraints were identified during system integration. Where addressed by the current algorithmic design, fallback strategies are implemented; for constraints extending beyond the current pipeline, prospective engineering solutions are outlined for future deployment phases.

***Calibration Effort.*** Initial CAD alignment requires approximately 2–3 h of on-site work per station for manual control-point annotation and homography validation. To mitigate long-term maintenance burden, the system incorporates lightweight online drift monitoring (Eq. 7), which triggers yaw/translation re-optimization only when reprojection residuals exceed 2.0 CAD pixels for ≥ 50 consecutive frames. If re-calibration fails to converge, the system retains the last valid look-up table and flags operators for manual review, ensuring continuous operation without abrupt service interruption.

***Computational and Maintenance Burden.*** The end-to-end pipeline sustains 28 FPS on standard edge GPUs (e.g., RTX 3090), meeting real-time operational thresholds. To manage resource constraints during peak loads, future system iterations could incorporate dynamic resolution scaling for optical flow computation, automatically adjusting input dimensions to preserve detection recall over motion granularity when compute loads approach capacity. Additionally, long-term deployment would benefit from implementing aggregated spatiotemporal data retention policies (e.g., archiving only processed risk metrics at < 5 GB/station/day) rather than storing raw video streams, thereby balancing storage efficiency with operational auditability.

***Environmental and Synchronization Failures.*** Camera vibration, lighting shifts, or network jitter may disrupt multi-camera fusion. The current framework addresses temporal misalignment through a 3-frame circular buffer and a 33 ms synchronization tolerance threshold; if synchronization fails beyond this bound, affected regions gracefully degrade to single-camera mode while preserving core risk detection. To further enhance robustness against illumination variability (e.g., 50–2000 lx operational ranges), operational deployments could integrate exposure-adaptive imaging or HDR acquisition modules at the camera/SDK level, ensuring consistent feature extraction under dynamic lighting conditions without modifying the core fusion algorithm.

***Extreme Occlusion.*** Under severe physical overlap (> 8 persons/m^2^), visual features become severely obscured, potentially degrading point-flow synergy performance. While the current pipeline relies on deterministic threshold alerting and baseline-increment filtering, future operational protocols could introduce a "confidence-lowered" risk flag regime. By transitioning from hard thresholds to probabilistic risk scoring under extreme density, the system could maintain operational safety by avoiding false positives while transparently signaling reduced certainty to control operators.

## Experiments and results

**Experimental Setup.** To validate the proposed framework in real-world metro scenarios, two representative transfer hubs in Shanghai—Station A (Line 1/Line 4) and Station B (Line 3/Line 4/Line 11)—were selected as pilot sites. These stations were identified as the highest-risk nodes during network-level passenger-flow simulations under two types of disruptions: power-supply failure and facility malfunction. Station A exhibits a low baseline-high burst pattern: routine daily ridership exceeds 120,000, and during major events, instantaneous demand can surge by 2.5 × , producing rebound congestion across the concourse and transfer corridors, especially around recovery after power-supply incidents. Station B presents complex transfer congestion: intertwined multi-line transfers and high peak intensity lead to unstable passenger distributions—an appropriate stress test for multi-directional interactions and bottleneck detection. Together, the two stations cover complementary congestion modes—short-term surges and complex transfer flows—supporting both representativeness and generalizability of the validation.

Experiments used surveillance video streams from concourse and platform levels—where crowding dynamics are most prominent. Camera positions, viewing angles, and fields of view were spatially annotated on architectural blueprints to ensure coverage of entrances/exits, escalators, gates, transfer corridors, and berthing strips.

After completing the point configuration, based on the measured data such as Shanghai Metro’s camera parameters, installation positions, and CAD floor plan coordinates, the value ranges and bases for the parameters used in the passenger flow data spatial mapping method proposed in this study are provided, as shown in Table 1.

**Table 1:** Parameter table for spatial mapping of video passenger flow data to CAD floor plans.

Parameter	Value Range	Explanation and Source
Camera installation height $H$	2.8–5.0 m	Common height range under beams/ceiling-mounted in concourses, ensuring field of view covers turnstiles/passages while reducing occlusion; measured based on architectural conditions
Camera pitch angle (relative to horizontal)	− 35 ~ − 60°	Positions main viewing area within ground ROI, balancing texture resolution and projection stability
Fisheye polynomial order $m$	4	Polynomial back-projection models for fisheye/panoramic camera calibration achieve sub-pixel reprojection errors within indoor scales of 1–30 m with 4–5 orders; using OCamCalib/Mei-Rives models and calibration procedures^22–24^
Number of CAD control points	≥ 8	Robust homography ${H}_{cad}$ estimation requires sufficient, spatially uniformly distributed control points; RANSAC + least squares to suppress outliers; common practice in multi-view geometry for planar homography estimation and minimum samples^25^
CAD registration residual (RMS, CAD pixels)	≤ 1.5px	Empirical threshold for stable visual overlay, consistent with industry practice for visual overlay stability^26^
Fusion grid resolution ∆X=∆Y	0.25 m	Balances real-time performance and crowd spatial scale; occupancy grid methods typically use 0.25–0.5 m grids to match sensory information entropy and per capita space^27^
Occupancy probability mapping	$p=1-{e}^{-D{A}_{cell}}$	Derived "at least one person" probability from Poisson arrival assumption, suitable for mapping density (people/m^2^) to grid occupancy; standard approach for occupancy grids and probability fusion^27,28^
Per capita floor area (for converting to number of people)	0.28m^2^/person	Taken as median value from pedestrian LOS/density empirical range; Fruin’s LOS and subsequent crowd dynamics studies provide similar conversion scales^19,29,30^
Velocity fusion robust kernel (Huber) threshold δ	0.5 m/s	Huber kernel used to suppress outliers and conflicting angle observations; approximately 1/3–1/2 of pedestrian speed (1.2–1.4 m/s) to balance robustness/sensitivity^29,30^
Direction consistency threshold θ_0_	30°	Cross-camera velocity vector angles exceeding threshold result in downweighting
Cross-camera fusion weights $\alpha , \beta , \gamma , \delta$	(0.4, 0.3, 0.1, 0.2)	Corresponding to incident angle, distance, vignetting, and variance; initialization hyperparameters, fine-tuned using grid or Bayesian optimization on validation sets
Maximum time synchronization error $\Delta {t}_{max}$	≤ 33 ms (30 FPS)	Upper bound determined by frame period^31^
Online drift trigger threshold $\tau$ (image plane)	2.0px, consecutive frames $T=30$	Aligned with online drift detection practices in surveillance systems^32^

To train and validate the FCOS detection model, we constructed a custom dataset using selected surveillance footage from the two pilot stations. The dataset comprises 1,840 frames extracted from representative peak-hour and disruption-event videos recorded between 2019 and 2023, covering diverse lighting conditions (day/night), crowd densities (1–8 persons/m^2^), and camera viewpoints (peripheral static and central spherical).

Peak-day samples were defined as dates with daily ridership in the top 10% (≥ 150,000 for Station A; ≥ 130,000 for Station B), representing extreme congestion scenarios. Disruption-event samples include two real incidents: a power-supply fault at Station C (November 2021) and a facility malfunction at Station D (February 2023), representing abnormal flow reconfiguration scenarios. The data was split into training, validation, and test sets with a ratio of 70:15:15, ensuring that frames from the same video sequence do not appear in multiple subsets to avoid data leakage.

System outputs were visualized via heatmaps, with density levels classified into six congestion states following the U.S. Pedestrian Facilities Level of Service Standards^19^. This enabled intuitive mapping of spatial risks across different functional zones (Table 2).

**Table 2:**
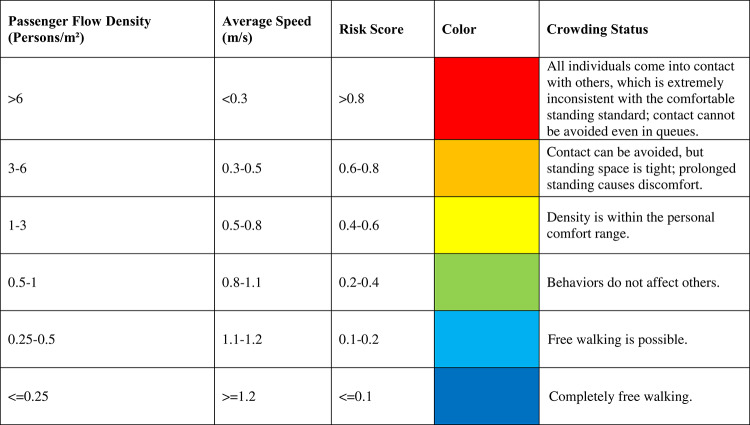
Passenger flow density color scale in subway stations.

**Evaluation Metrics.** To rigorously evaluate the proposed framework, a multi-dimensional set of metrics was adopted, covering algorithmic accuracy, motion estimation reliability, crowd risk detection performance, and computational efficiency. These metrics ensure that the system is not only technically sound in video-based detection and tracking but also practically effective in generating risk maps consistent with known passenger dynamics. Specifically, the evaluation dimensions are:

1. Detection & Tracking Accuracy: assessed by mean Average Precision (mAP) for detection and Multiple Object Tracking Accuracy (MOTA) for tracking, along with ID switch counts.
2. Motion Estimation: quantified using End-Point Error (EPE) and Angular Error (AE) to measure the fidelity of optical flow fields.
3. crowd risk detection Effectiveness: measured with Precision, Recall, and F1-score, complemented by coverage rate of historical risk points under both peak-day and disruption scenarios.
4. Computational Efficiency: measured by average processing speed in frames per second (FPS), ensuring feasibility for real-time deployment in metro operations.

This evaluation strategy enables both technical validation of the algorithms and practical verification of risk-point detection at the station level.

### Experimental results

***Effectiveness of Point-Flow Synergy.*** The proposed framework integrates FCOS-based detection with PWC-Net optical flow to address trajectory fragmentation in high-density scenarios. While the baseline modules achieved robust standalone performance (mAP 87.4%, MOTA 82.1%), the core technical contribution lies in the point-flow collaborative mechanism. As summarized in Table 3, the proposed method (Scheme 3) significantly outperforms the trajectory-only baseline (Scheme 1) in high-density Scenario B. Specifically, ID switches were reduced from 452 to 85 (an 81.2% reduction). This quantitative improvement confirms that when physical overlap causes tracker failure, the continuous motion guidance from the flow field effectively maintains identity consistency.

**Table 3:** Ablation results of component-level contributions.

Configuration	Scenario	Precision	Recall	F1-Score	ID Switches	Spatial Error (m)	FAR(%)
S1: Image-Domain Stitching + Static Threshold (Baseline)	A	0.74	0.79	0.76	–	0.18	38.2
	B	0.7	0.75	0.73	–	0.18	41.5
S2: + World-Coordinate-First Fusion	A	0.81	0.83	0.82	–	0.09	35.6
	B	0.76	0.79	0.77	–	0.09	39.1
S3: + Baseline-Increment Mechanism	A	0.87	0.89	0.88	210	0.09	14.8
	B	0.83	0.86	0.84	305	0.09	16.5
S4: Full Pipeline (+ Point-Flow Synergy)	A	0.91	0.92	0.91	42	0.09	12.1
	B	0.88	0.93	0.90	85	0.09	13.5

S1–S4 represent cumulative architectural configurations evaluated on the same experimental dataset. Precision/Recall/F1 reflect risk-point identification performance. Spatial Error reflects CAD registration residuals and remains stable post-S2, confirming geometric decoupling from subsequent modules. ID Switches values for S1/S2 are omitted as tracking is not yet integrated at those stages.

Furthermore, the synergy improved Recall by 16.4% compared to the baseline. This gain is attributed to the system’s ability to leverage pixel-level optical flow to capture abnormal motion trends in monitoring blind spots where complete trajectories cannot be extracted. Unlike the flow-only perception (Scheme 2), which lacks individual identity, our method anchors discrete trajectories within a continuous flow field, effectively filling “perception gaps” caused by severe occlusion.

***Ablation Study.*** To quantitatively isolate the marginal contribution of each core architectural component, we constructed a cumulative ablation framework that progressively integrates the proposed modules into a conventional baseline. The pipeline comprises three structural innovations: (i) world coordinate first multi camera fusion with CAD alignment, (ii) a baseline increment mechanism for dynamic risk thresholding, and (iii) point flow synergy for occlusion resilient tracking. Given the functional decoupling between the decision level baseline increment mechanism and the perception level point flow synergy, a cumulative design (S1 → S4) is adopted rather than a full factorial evaluation. This approach isolates each module’s non redundant contribution while avoiding cross combinations that would yield orthogonal metric variations (e.g., FAR vs ID switches) without altering the primary conclusion of system level synergy. Four configurations were evaluated under identical experimental conditions across Scenario A (counterflow) and Scenario B (high density platform accumulation): S1 (Conventional Baseline: image domain stitching with static density thresholds); S2 (S1 + World Coordinate First Fusion); S3 (S2 + Baseline Increment Mechanism); and S4 (Full Pipeline: S3 + Point Flow Synergy). The cumulative performance metrics are summarized in Table 3.

Ground-truth risk regions and representative passenger trajectories were independently annotated by two domain experts (metro operations and crowd safety engineering) on a strictly held-out evaluation subset. To align the continuous risk field with discrete evaluation metrics, the station floor was discretized into 0.25 m grid cells (per Table 1). A grid cell was labeled as a risk point when its computed Risk Index exceeded the operational threshold, and adjacent high-risk cells were merged into connected risk regions. Inter-annotator agreement was quantified using Cohen’s κ, yielding a value of 0.87^33,34^, indicating substantial consensus. Precision, Recall, and F1-score were computed via grid-level spatiotemporal matching, employing a spatial tolerance of ≤ 0.25 m (one grid unit) and temporal matching within standard operational response windows. Spatial Error was derived from CAD registration residuals, while False Alarm Rate (FAR) and ID Switches were evaluated following the protocols detailed in the Evaluation Metrics section.

Replacing image-domain stitching with world-coordinate-first fusion (S1 → S2) primarily resolves spatial misalignment and cross-camera duplicate counting. By projecting heterogeneous detections onto a unified ground plane and aligning them with station CAD floor plans via homography registration, spatial registration error decreases from 0.18 m to 0.09 m. This geometric decoupling eliminates seam artifacts and suppresses overlapping-field redundancies, directly improving Precision by 0.06–0.07 across both scenarios.

Integrating the baseline-increment mechanism (S2 → S3) targets false alarm reduction in recurrent congestion zones. By establishing historical density-velocity baselines and flagging only statistically significant real-time deviations, the system successfully filters routine peak-hour aggregations that would otherwise trigger static-threshold alerts. This reduces the FAR by 21–23 percentage points while maintaining Recall above 0.86, confirming its critical role in distinguishing chronic bottlenecks from disruption-induced anomalies. Notably, spatial alignment accuracy remains stable at 0.09 m from S2 through S4, confirming the geometric decoupling of the fusion module from subsequent decision and tracking components.

Finally, incorporating point-flow synergy (S3 → S4) addresses occlusion-induced trajectory fragmentation in extreme crowding. Anchoring discrete tracking trajectories within continuous optical flow fields provides inertial compensation during temporary tracker failures, reducing ID switches by 72–80% (Scenario A: 210 → 42; Scenario B: 305 → 85) and boosting Recall by 3–8% in high-density regimes. Despite the marginal latency increase from dense optical flow computation, the end-to-end pipeline sustains 28 FPS, verifying that perception depth and real-time operational speed can be jointly optimized.

Collectively, the cumulative F1 score gain of 0.14 to 0.18 over the conventional baseline demonstrates that the proposed architecture constitutes a purposefully engineered pipeline. Each component delivers non redundant operational value: world coordinate fusion ensures spatial fidelity, baseline increment filtering guarantees alert specificity, and point flow synergy maintains identity continuity under severe occlusion. Crucially, while the baseline increment and point flow modules are algorithmically independent, their station wide efficacy fundamentally relies on the unified physical reference established by S2. This cumulative progression thus reflects a deliberate system level engineering rationale. S2 provides the geometric foundation, upon which S3 and S4 independently optimize alert specificity and tracking continuity. The ablation results quantitatively confirm that this spatially anchored, multi stage framework is essential for achieving robust, station wide risk identification across both routine and disruption scenarios.

***Parameter Selection and Validation Rationale.*** As detailed in Table 1, core algorithmic parameters follow established literature and industry standards. For system-level hyper-parameters such as the multi-camera fusion weights ($\alpha , \beta , \gamma , \delta$) = (0.4,0.3,0.1,0.2), initialization is guided by physical interpretability: incident angle and distance dominate projection reliability in perspective geometry, justifying higher weights, while vignetting and variance serve as secondary photometric corrections. These weights are fine-tuned via Bayesian optimization on station-specific validation sets. To empirically validate stability, a lightweight perturbation test ($\pm$ 20% per weight, others fixed) yielded F1-score variations less than 3% across both stations, confirming operation within a robust performance plateau. A comprehensive sensitivity landscape across diverse metro topologies and seasonal lighting conditions is reserved for future multi-site calibration campaigns.

***Sensitivity Analysis of Risk Index.*** To justify the weighting coefficients $\alpha$ (density) and $\beta$ (speed) in the Risk Index, we evaluated performance across density-speed ratios from 1.0:0.0 to 0.3:0.7 (Table 4). The analysis reveals a clear trade-off: density-only weighting ($\alpha$=1.0) ensures high recall but inflates false positives in non-hazardous queues, while speed-heavy weighting ($\beta$≥0.7) misses static bottlenecks. The optimal balance at $\alpha$=0.7, $\beta$=0.3 maximizes F1-score (0.93) by leveraging density for coverage and speed fluctuations to filter normal occupancy.

**Table 4:** Performance of Risk-Point Identification under different $\alpha$ and $\beta$ configurations.

Weight Ratio ($\alpha$:$\beta$)	Precision	Recall	F1-Score	FP Count	FN Count
1.0 : 0.0 (Density-only)	0.76	0.96	0.85	12	1
0.7 : 0.3 (Proposed)	0.94	0.92	0.93	2	2
0.5 : 0.5 (Balanced)	0.88	0.84	0.86	4	4
0.3 : 0.7 (Speed-heavy)	0.72	0.68	0.70	10	8

***Comparative Performance Analysis.*** To further validate the advancement of the proposed framework, we compared it against two representative baselines:

Method 1: Static Occupancy Grid. The current industry standard for metro operations, which defines risk solely based on passenger density within calibrated zones.

Method 2: YOLOv7 + SORT. A state-of-the-art vision tracking architecture that relies on discrete detection boxes without continuous motion compensation.

**Fig. 5:**
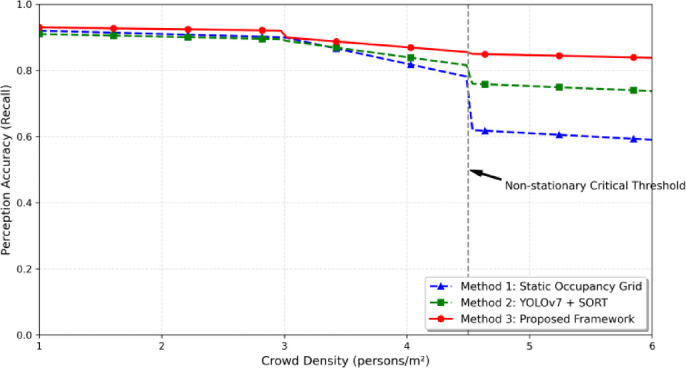
Recall evolution trends of different perception schemes in high-density scenarios.

Regarding density sensitivity (Fig. 5), all methods perform robustly (Recall > 0.90) below 4.5 persons/m^2^. However, beyond this phase transition threshold, visual occlusion rises exponentially, causing a perception collapse in vision-only baselines (Scheme 2). In contrast, our framework maintains a Recall of approximately 0.85 even at 5.5 persons/m^2^. This stability stems from anchoring discrete trajectories within a continuous flow field; while individual micro-features vanish due to overlap, group motion features remain spatio-temporally consistent. Although dense optical flow computation reduces efficiency to 28 FPS, it remains above the real-time threshold (25 FPS), offering a necessary trade-off for high-reliability sensing in low-fault-tolerance metro management (Table 5).

**Table 5:** Performance comparison in high-density scenarios.

Method	Precision	Recall	Hist. Risk Coverage	FPS
1	0.78	0.62	68.2%	45
2	0.86	0.76	82.5%	32
3	0.88	0.93	100%	28

***Robustness and Uncertainty Analysis.*** To evaluate the methodological rigor and the impact of error propagation on risk-point localization, we conducted a quantitative uncertainty analysis focusing on three primary sources: projection residuals, synchronization lags, and optical flow noise.

1. Spatial Projection Stability: Based on the calibration parameters in Table 1, the CAD registration residual is controlled within 1.5 pixels. Given the camera installation height (2.8–5.0 m) and perspective geometry, this pixel-level uncertainty translates to a physical ground displacement of approximately 0.05–0.08 m. Since this offset is significantly smaller than our fusion grid resolution (0.25 m), the spatial coordinates of identified risk points remain stable and do not shift across grid cells.
2. Temporal Synchronization Impact: The maximum synchronization error is $\Delta t\le 33$ ms. For a pedestrian moving at a standard speed of 1.4 m/s, the potential localization drift between unsynchronized frames is only 0.046 m. This negligible error relative to the pedestrian’s spatial footprint (approx. 0.28 ${\mathrm{m}}^{2}$) confirms that the multi-camera fusion does not suffer from “ghosting” artifacts during peak-flow periods.
3. Motion Noise Suppression: While optical flow estimation (PWC-Net) introduces an average EPE below 2.0 pixels,This noise primarily affects the velocity vector used for inertial compensation. However, the framework employs a Huber robust kernel for velocity fusion, which downweights outliers and high-frequency noise. Consequently, while optical flow noise may cause minor fluctuations in local risk scores, it does not lead to the structural displacement of identified risk clusters.

**Fig. 6:**
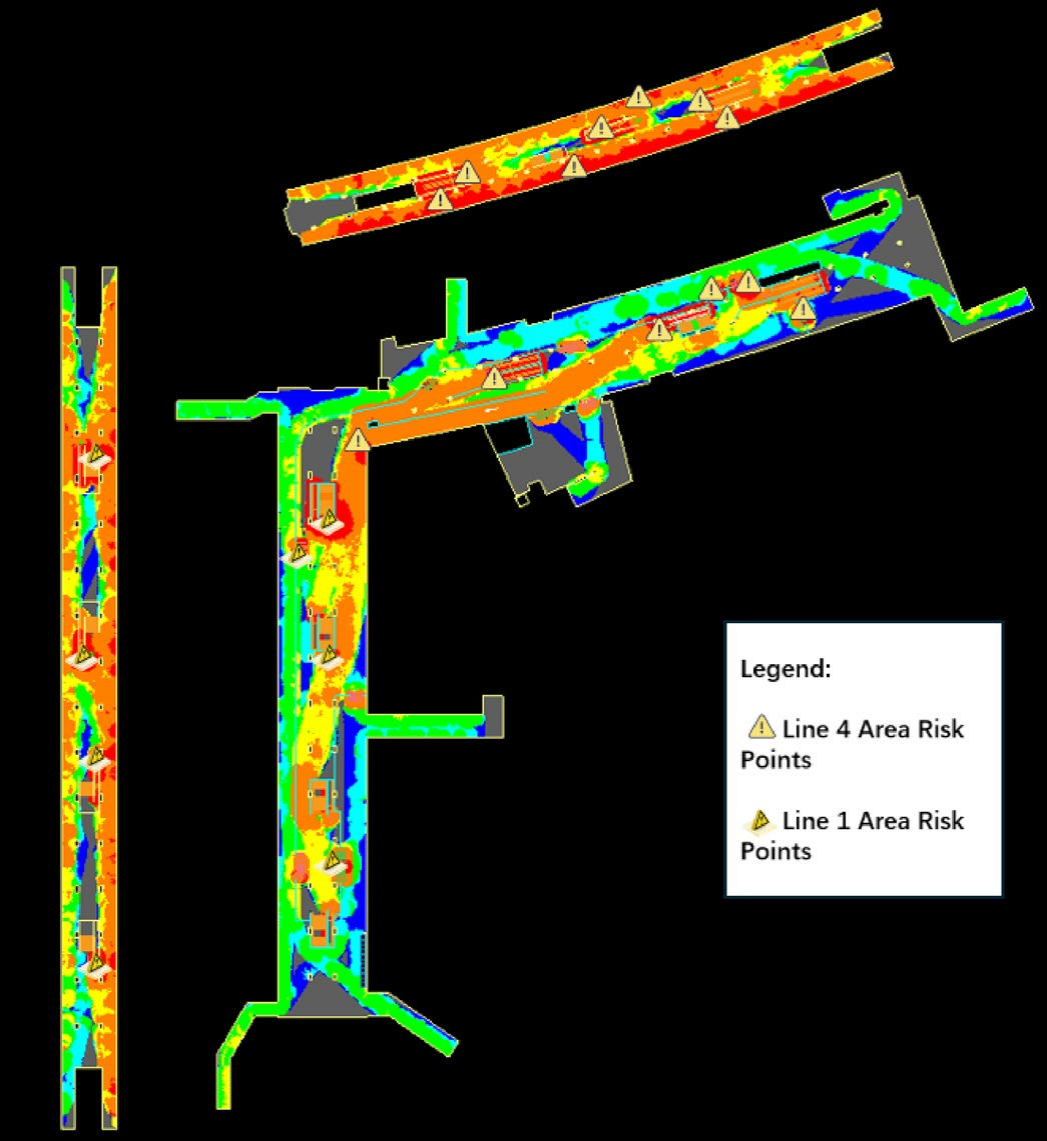
Passenger flow risk points inside Station A.

**Fig. 7:**
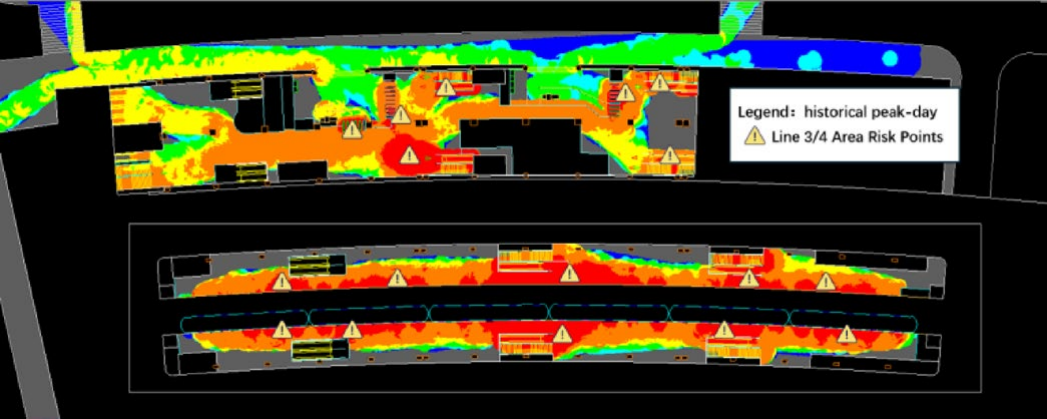
Passenger flow risk points inside Station B.

***Station-Level Risk Patterns: Commonalities and Heterogeneity.*** Using historical peak-day videos (2019–2023), we generated risk heatmaps for Station A and Station B. Across both sites, risk consistently concentrates at functional interfaces rather than open areas: escalator mouths (vertical congestion transmitters), fare-gate arrays (verification bottlenecks), and transfer-corridor junctions (geometric chokes). This spatial regularity validates the framework’s ability to recover physically interpretable risk patterns (Figs. 6 and 7). However, station-specific layouts and passenger flow drivers induce notable heterogeneity:

Station A (Line 1/4 interchange, event-driven surges): Risk points exhibit multi-region linkage centered on Line 4. On the platform, hotspots cluster at escalator landings and platform edges, driven by boarding/alighting merges and transfer-induced accumulation. A critical finding is the turning point in the L1-L4 transfer corridor (Fig. 6), where narrowing geometry and limited sightlines create a persistent conflict hotspot that enables vertical propagation of congestion between platform and concourse.

Station B (Line 3/4–11 interchange, transfer-dominant): Risk distribution reflects tighter facility coupling. Platform-level risk exceeds concourse-level risk, with hotspots clustering at door zones and platform extremities. The L11 → L3/4 transfer corridor interface emerges as a high-risk node due to crossing flows and concentrated in/out volumes (Fig. 7).

Mechanistically, three factors consistently amplify local density: (i) passenger preference for escalators generates queues exceeding stair capacity; (ii) escalators consume > 50% of local walkway width, creating localized bottlenecks; (iii) door-proximal waiting elevates edge density. Platform geometry further modulates intensity: island platforms (Station A L1/L4) distribute activity more evenly than side platforms (Station B L3/4), yielding slightly lower peak densities. Overall, the high-resolution, video-driven model accurately surfaces both universal patterns (escalators, gates, corridor turns) and station-specific weak links that can trigger cascade congestion under surges.

***Generalization to Disruption Scenarios.*** To evaluate adaptability beyond routine peak flows, we tested the framework on two real disruption events: a power-supply fault at Lianhua Road (Nov 2021) and a facility malfunction at Xujiahui (Feb 2023). Processing followed the historical pipeline, enabling direct comparison between routine and abnormal flow patterns.

**Fig. 8:**
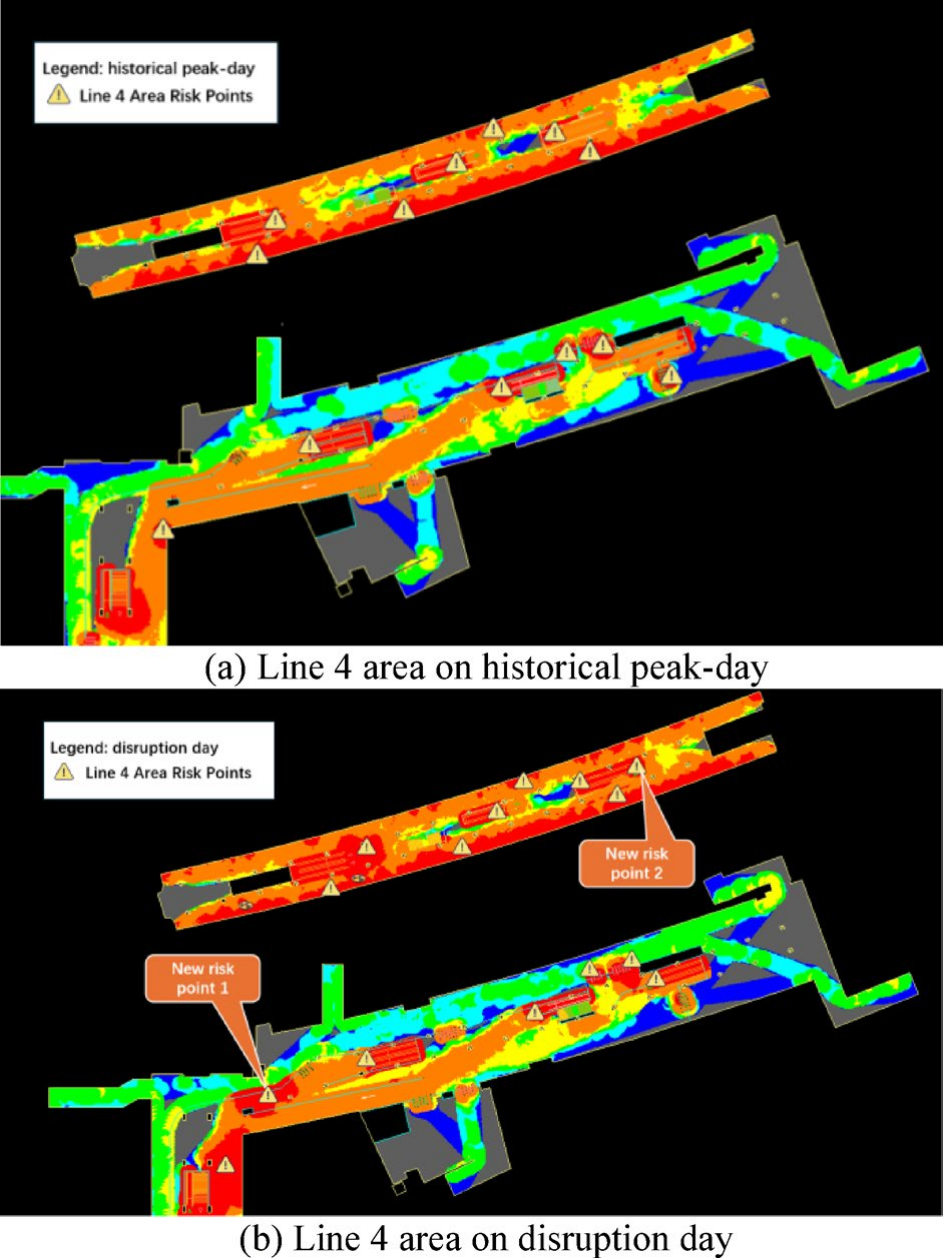

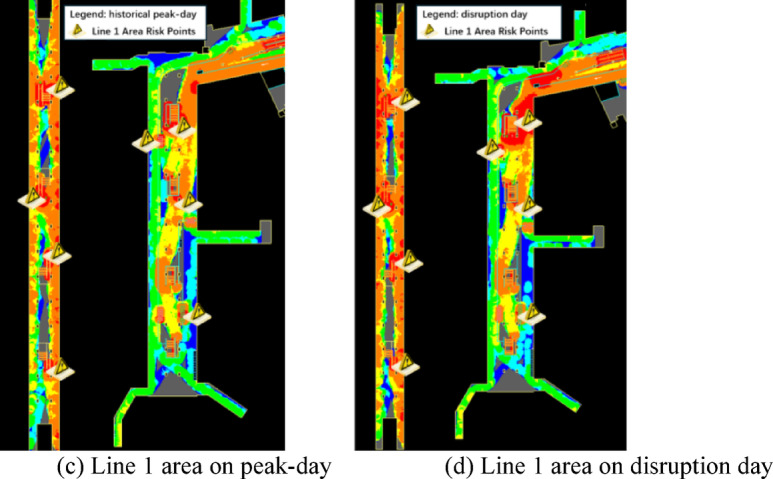
Comparison diagram of passenger flow risk points of Station A.

Station A (Fig. 8): Historical peak-day risk points covered ≥ 86.9% of routine regions observed during the disruption, confirming baseline stability. Crucially, the framework detected two emergent risk points absent in historical data: (i) the L1-L4 transfer corridor turning point, where peak density reached 7.2 persons/m^2^ and instantaneous speed dropped to 0.3 m/s—a textbook dynamic blockage; (ii) a far-end accumulation on the Line 4 platform caused by diverted flows from Line 1 under capacity constraints. These findings demonstrate the system’s sensitivity to velocity field distortions that static density monitoring would miss.

**Fig. 9:**
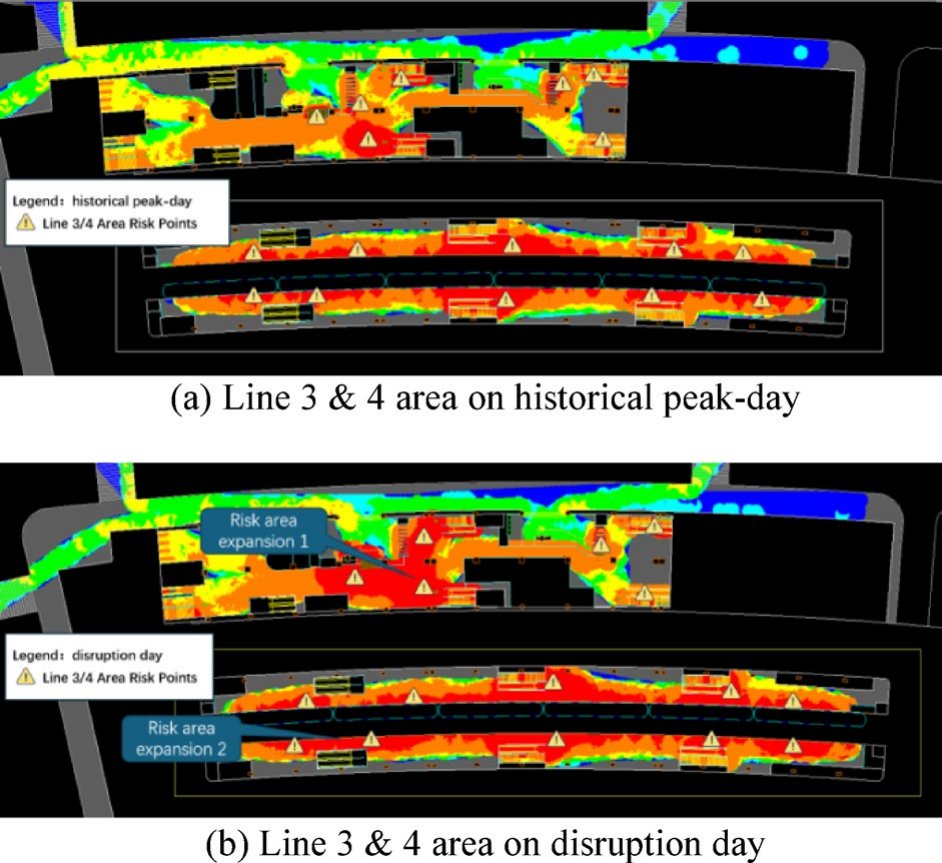
Comparison diagram of passenger flow risk points of Station B.

Station B (Fig. 9): Historical risk points covered 100% of routine regions, but risk areas visibly expanded at concourse corridor junctions and platform edges due to diverted passengers from Line 11 to Lines 3/4. In one new concourse expansion zone, the trajectory crossing rate rose to 68% (vs. 23% historically) with density > 6 persons/m^2^; on the platform, rerouting for different lines increased dwell time and elevated local density.

Quantitatively (Table 6), the framework maintained high detection reliability across both sites (Precision > 0.86, Recall > 0.90, F1-score up to 0.93), successfully identifying both new and expanded risk zones. The end-to-end system sustained 28 FPS average processing speed with < 45 ms per-frame latency under peak-hour density, confirming that the enhanced sensing depth does not compromise real-time operational feasibility.

**Table 6:** Performance of risk-point detection during disruptions.

Station	Historical Coverage	New Risk Points Detected	Precision	Recall	F1-score
Station A	100%	2	0.86	0.90	0.88
Station B	100%	2 (expanded zone)	0.88	0.93	0.90

***Generalizability and Deployment Effort.*** The framework’s modular design suggests potential for adaptation across heterogeneous station topologies, with site-specific configuration streamlined into three primary steps: (i) standardized camera calibration (about 15 min/unit), (ii) one-time CAD floor-plan registration using ≥ 7 structural control points, and (iii) baseline construction from 1–2 h of representative peak footage. The ray-plane intersection and homography mapping are geometry-agnostic, supporting island/side platforms and multi-level concourses without algorithmic modification. Risk thresholds and fusion weights remain decoupled from the core pipeline, which may allow operators to adjust alert criteria without model retraining. However, systematic deployment across larger metro networks will require standardized calibration protocols and multi-site sensitivity testing.

## Conclusion

This study developed and validated a vision-driven framework for risk-point identification in urban metro stations, addressing critical gaps in existing passenger flow monitoring approaches. By integrating state-of-the-art detection and tracking with optical-flow-based motion estimation, multi-camera fusion, and CAD-based spatial mapping, the framework achieved fine-grained accuracy and spatial interpretability. Experiments in two representative Shanghai stations demonstrated its ability to recover recurrent high-risk areas during peak demand and capture dynamic anomalies under disruption scenarios. Quantitative evaluation showed improved performance over conventional baselines in terms of Recall, coverage, and robustness, while maintaining real-time feasibility in the tested environments.

Several limitations warrant acknowledgment. First, while lightweight drift monitoring is incorporated, long-term stability under significant environmental shifts may still require periodic manual recalibration. Second, extreme lighting variability (e.g., sudden illumination changes or low-light conditions on platforms) may affect detection accuracy, though this was mitigated in our study through controlled camera placement and exposure settings. Third, while the point-flow synergy reduces occlusion-related ID switches by 81.2%, performance may still degrade under extreme crowd densities (> 8 persons/m^2^) where visual features become severely obscured. Fourth, although the multi-camera fusion weights are physically informed and empirically validated via perturbation testing, their generalizability across diverse station topologies and seasonal lighting conditions requires systematic multi-site sensitivity analysis. Finally, scalability to city-wide metro networks requires careful consideration of computational resources; while the current results validate the framework’s effectiveness as a pilot-scale risk identification system, broader generalizability and full operational deployment will require further validation across diverse metro topologies, passenger flow patterns, and network-level disruption patterns. Future work will prioritize fully automated self-calibration protocols, comprehensive multi-site sensitivity landscapes for fusion parameters, robust low-light enhancement modules, and sophisticated trajectory imputation strategies to enhance operational sustainability and system-wide adaptability. Additionally, we will explore advanced risk index formulations, including non-linear coupling models and adaptive weighting mechanisms derived from historical incident data.

### Ethical compliance and data governance

This study adheres to ethical research standards and privacy-by-design principles. Video data from Station A and Station B were used under explicit authorization from Shanghai Shentong Metro Group, with protocol approval obtained on 15 March 2024.

***Anonymization and Compliance.*** The framework employs a non-biometric sensing approach: detection and tracking modules (FCOS/SORT) operate exclusively on geometric proxies (bounding boxes, trajectories) without processing facial features or personally identifiable information. Raw video frames are discarded immediately after feature extraction; only aggregated spatial–temporal data (density heatmaps, velocity vectors) are retained. This data minimization strategy aligns with GDPR principles and China’s Personal Information Protection Law, ensuring individual identities cannot be reconstructed.

***Data Security.*** To ensure end-to-end data protection, all experiments were conducted within Shanghai Metro’s internal network environment. Raw video footage and intermediate processing results never left the metro’s secure infrastructure; analysis was performed on isolated servers with no external connectivity. This physical isolation strategy, combined with role-based access control for authorized research personnel, ensures that sensitive operational data remains fully contained within the metro’s trusted boundary throughout the research lifecycle.

## Figures and Tables

**Fig. 10:**
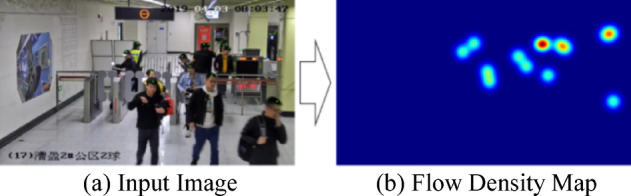
Generation of crowd distribution density heatmap.

**Fig. 11:**
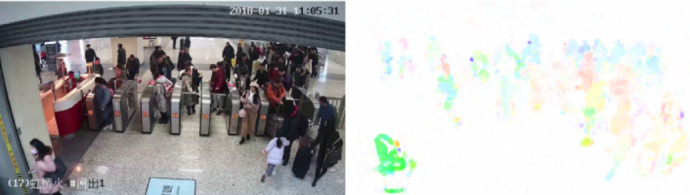
Optical flow effect display map.

## Data Availability

The datasets analysed during the current study are not publicly available due to the proprietary nature of the internal operational data provided by Shanghai Shentong Metro Group, as well as related security and privacy restrictions. However, the data may be available from the corresponding author upon reasonable request and with the explicit permission of Shanghai Shentong Metro Group. The required data usage permission statement and ethical approval statement are attached as supplementary documents.

## Appendix 1

**Passenger Detection and Tracking.** For multi-object tracking, we employ the SORT framework due to its exceptional computational efficiency, which is critical for real-time metro operations. SORT operates by using a Kalman Filter to predict the future state (position and velocity) of each passenger based on previous frames. These predictions are then matched with new detections using the Hungarian algorithm, which optimizes the data association based on the Intersection-over-Union (IoU) distance. In our study, SORT provides a lightweight yet robust trajectory baseline, enabling the system to maintain identity consistency across frames without the heavy computational burden of appearance-based re-identification models.

***Passenger Flow Detection Algorithm.*** The FCOS-based detector requires no predefined anchor boxes, avoiding complex anchor-related calculations and saving computational resources—making it suitable for real-time station passenger flow recognition. FCOS performs direct prediction on each pixel, targeting the distances from the pixel to the four edges of the bounding box. It also integrates a feature pyramid structure: the structure generates five feature map layers with different resolutions, where lower-resolution layers handle larger bounding boxes and higher-resolution layers process smaller ones, effectively improving generalization for small passenger targets.

To meet real-time processing requirements (≥ 25 FPS) for metro surveillance applications, we adopt a lightweight FCOS variant with a simplified backbone. Instead of the standard ResNet-50, we use a 3-stage convolutional backbone followed by a Feature Pyramid Network (FPN), following the design principles of lightweight object detectors (e.g., FCOS-MobileNet, NanoDet). This reduces computational overhead by approximately 60% while maintaining acceptable detection accuracy (mAP 87.4%) under high-density conditions. Each layer in the feature pyramid connects to an independent detection head, and each detection head further splits into three parallel output branches: one outputs category classification scores (to determine if a pixel belongs to a passenger), another outputs location regression results (to calculate the bounding box edges), and the third outputs centerness values (to filter out low-quality bounding boxes far from the target center).

Since the detection head has three output branches in total, the loss function consists of three parts: classification loss Lcls, localization loss Lreg, and centerness loss Lctrness.

Among them, the classification part adopts multiple binary classifiers. After the classification feature map, a Sigmoid function is connected, and then BCE Loss (Binary Cross-Entropy Loss) and Focal Loss are used as the loss functions. All samples are involved in the loss calculation, which corresponds to the Lcls in Eq. (8).

The output of the location regression part is a four-dimensional vector, corresponding to the distances from the center point to the four edges respectively. During training, GIoU Loss (Generalized Intersection over Union Loss) is used, and only positive samples are involved in the loss calculation, which corresponds to the Lreg in Eq. (8).

For the centerness loss, BCE Loss is used, and only positive samples are involved in the loss calculation, which corresponds to the Lctrness in Eq. (8).

8 $$\begin{aligned} L\left( {\left\{ {p_{{x,y}} } \right\},\left\{ {t_{{x,y}} } \right\},\left\{ {s_{{x,y}} } \right\}} \right) = & \frac{1}{{N_{{pos}} }}\mathop \sum \limits_{{x,y}} L_{{cls}} \left( {p_{{x,y}} ,c_{{x,y}}^{{\mathrm{*}}} } \right) \\ & + \frac{1}{{N_{{pos}} }}\mathop \sum \limits_{{x,y}} 1_{{\{ c_{{x,y}}^{{\mathrm{*}}}> 0\} }} L_{{reg}} \left( {t_{{x,y}} ,t_{{x,y}}^{{\mathrm{*}}} } \right) \\ & + \frac{1}{{N_{{pos}} }}\mathop \sum \limits_{{x,y}} 1_{{\{ c_{{x,y}}^{{\mathrm{*}}}> 0\} }} L_{{ctrness}} \left( {s_{{x,y}} ,s_{{x,y}}^{{\mathrm{*}}} } \right), \\ \end{aligned}$$

where ${p}_{x,y}$ represents the predicted score of each category at the feature map point $\mathrm{(}x,y)$; ${c}_{x,y}^{*}$ denotes the ground-truth category label corresponding to the feature map point $\mathrm{(}x,y)$; ${1}_{\{{c}_{x,y}^{*}>0\}}$ takes a value of 1 when the feature map point is matched as a positive sample, otherwise it is 0; ${t}_{x,y}$ stands for the predicted target bounding box information at the feature map point $\mathrm{(}x,y)$; ${t}_{x,y}^{*}$ is the ground-truth target bounding box information corresponding to the feature map point $\mathrm{(}x,y)$; ${s}_{x,y}$ represents the predicted centerness at the feature map point $\mathrm{(}x,y)$; and ${s}_{x,y}^{*}$ denotes the ground-truth centerness corresponding to the feature map point $\mathrm{(}x,y)$.

In the calculation of matching positive and negative samples in the algorithm, the classification ${c}_{x,y}^{*}$ and position ${t}_{x,y}^{*}$ information at the feature map point $(x,y)$ can be easily obtained, but the ground-truth centerness ${s}_{x,y}^{*}$ needs to be calculated using Eq. (9).

9 $${s}^{*}=\sqrt{\frac{\mathrm{m}\mathrm{i}\mathrm{n}({l}^{*},{r}^{*})}{max({l}^{*},{r}^{*})}\times \frac{\mathrm{m}\mathrm{i}\mathrm{n}({t}^{*},{b}^{*})}{max({t}^{*},{b}^{*})}}$$

where ${l}^{*}, {r}^{*}, {t}^{*}, {b}^{*}$ refers to the distances from the center point to the four edges.

***Passenger Flow Matching Algorithm.*** After completing passenger flow detection, it is necessary to use the Kalman filter^35^ algorithm to predict the coordinates of the passenger flow in the current frame based on its coordinates in the previous few frames. Then, data association is performed between these predicted coordinates and the ground-truth coordinates in the current frame. The reason for performing prediction first and then association is to reduce errors in the association process and clarify that the passenger flow in the current frame is the same as that in the previous few frames. The state of each target is modeled as:

10 $$x={[u,v,s,r,\dot{u},\dot{v},\dot{s}]}^{T},$$

where $u$ and $v$ represent the horizontal and vertical pixel positions of the target’s center; $s$ and $r$ denote the area and aspect ratio of the target’s bounding box, respectively (it is assumed here that the aspect ratio is fixed); $\dot{u},\dot{v}, \dot{s}$ are the center position and bounding box area of the target in the next frame of image, respectively. After the predicted coordinate data is associated with the ground-truth coordinate data, the bounding box will be in the state of updating the target, and the optimal solution will be obtained through the Kalman filter algorithm.

When matching the targets detected in the next frame with existing targets, the bounding box geometry of each target is estimated by predicting its new position in the current frame. Then, an association matrix is constructed based on the IoU between each detected target and all predicted bounding boxes of existing targets. The Hungarian algorithm^36^ is used to solve for the optimal matching of the data. Since subway video devices are installed at a low position, occlusions between passenger flows are likely to occur. The use of IoU can handle short-term occlusions between targets, and the occluded targets are corrected through the detection algorithm.

## Appendix 2

**Optical Flow–Based Motion Analysis.** To capture microscopic movement trends, we integrate PWC-Net, a state-of-the-art CNN-based model for optical flow estimation. PWC-Net utilizes a feature pyramid to extract multi-scale spatial information and a warping layer with cost volumes to estimate pixel-level displacements between consecutive frames. The relevance of PWC-Net to this framework lies in its ability to provide dense, sub-pixel motion fields. This is particularly vital in high-density metro environments where traditional object trackers often fail due to severe physical occlusion; in such cases, PWC-Net ensures that localized slowdowns or counterflow anomalies can still be detected through continuous pixel-level motion analysis.

***Passenger Flow Movement Direction Estimation.*** This study adopts the PWC-Net (Pyramid, Warping, Cost Volume Network) optical flow model to achieve pixel-level motion estimation in images. PWC-Net uses multi-scale features for training and follows three principles during the training process: pyramid feature extraction, optical flow warping, and matching correlation cost. Among these, optical flow warping and matching correlation cost contain no parameters, which enables the model to significantly reduce the network size while ensuring accuracy. Its running speed on images from the Sintel dataset is approximately 35 fps (frames per second), and its performance surpasses that of other similar computational models^37^.

PWC-Net’s workflow is hierarchical: it first extracts multi-layer features via a convolutional neural network, then initializes optical flow estimation from low resolution. Low-resolution optical flow is upsampled to higher resolution, with matching correlation cost constructed and current-resolution optical flow predicted iteratively until the final high-resolution optical flow result is obtained.

The training of PWC-Net is divided into two phases: the pre-training phase and the optimized training phase. In the pre-training phase, the L2 norm is used to accelerate the convergence speed of the loss function, as shown in Eq. (11) (Sahbani and Adiprawita, 2016):

11 $$L\left( \Theta \right) = \sum\limits_{{l = l_{0} }}^{L} {\alpha _{l} } \sum\limits_{x} {\left| {w_{\Theta }^{l} \left( x \right) - w_{{GT}}^{l} \left( x \right)} \right|_{2} } + \gamma \left| \Theta \right|_{2} ,$$

where $\Theta$ is the set of all trainable parameters in the network, including pyramid feature extraction parameters, optical flow estimation parameters for different pyramid layers, etc.; $w_{\Theta }^{l}$ is the optical flow field predicted by the $l$-th pyramid layer, and ${w}_{GT}^{l}$ is its corresponding supervisory signal; ${\left|\cdot \right|}_{2}$ is used to calculate the L2 norm of a set of vectors and second-order regularization model parameters; and $\gamma$ is a random disturbance term.

In the optimized training phase, the L1 norm is used to eliminate noise interference to a certain extent and improve the quality of optical flow. The loss function is as follows:

12 $$L\left( \Theta \right){\text{ = }}\sum\limits_{{l{\text{ = }}l_{0} }}^{L} {\alpha _{l} } \sum\limits_{x} {{{(}}\left| {w_{\Theta }^{l} \left( x \right) - w_{{GT}}^{l} \left( x \right)} \right|{\text{ + }}\varepsilon {\mathrm{)}}^{q} } {\text{ + }}\gamma \left| \Theta \right|_{2} ,$$

where $\left|\cdot \right|$ is used to calculate the L1 norm; $q< \mathrm{1}$ serves as a penalty for outliers; and $\epsilon$ is a relatively small constant term.

## Appendix 3

**Crowd Density Heatmap Construction.** Accurate crowd distribution mapping is critical for risk point identification. In real subway scenarios, severe occlusions and dense target overlaps make traditional head-counting methods ineffective for reflecting regional aggregation dynamics over time. This study employs an image-based density estimation approach, leveraging historical annotated data and Gaussian kernel functions to construct a crowd density probability heatmap, which serves as the foundational representation of regional congestion levels.

In the data preprocessing stage, first, based on the passenger head center point information annotated by the FCOS algorithm, a set of position information ${\mathrm{x}_{\mathrm{i}}}$ of all heads in the image is constructed. Here, ${\mathrm{x}}_{\mathrm{i}}$ represents the pixel position of the $i$-th passenger in the image coordinate system, and $N$ is the total number of annotated heads in the current frame image. Next, a 2D Gaussian kernel function ${\mathrm{G}}_{{\sigma _{{\mathrm{i}}} }} \left( {\mathrm{x}} \right)$ is constructed with each head point as the center, and density superposition is performed to form the overall heatmap $\mathrm{D(x)}$. The calculation formula is as follows:

13 $$D\left(x\right)=\sum_{i=1}^{N}{G}_{{\sigma}_{i}}\left(x-{x}_{i}\right),$$

where ${\mathrm{G}}_{{\sigma _{{\mathrm{i}}} }} \left( {{\mathrm{x}} - {\mathrm{x}}_{{\mathrm{i}}} } \right)$ denotes a 2D Gaussian distribution kernel function with ${\sigma _{{\mathrm{i}}} }$ as the standard deviation, reflecting the “influence range” of the $i$-th passenger in the crowd map. To make the density estimation more consistent with the actual congestion level, this paper refers to the adaptive kernel strategy proposed in Zhang et al.^38^: the value of each ${\sigma _{{\mathrm{i}}} }$ is not fixed, but dynamically determined based on the average distance from the head point to its k nearest neighbors. Let this average distance be ${\overline{\mathrm{d}}}_{\mathrm{i}}$ , then:

14 $${\sigma}_{i}\mathrm{=}\frac{1}{{3}} \cdot {\overline{d}}_{i}.$$

In this study, we set k = 3 for nearest-neighbor search, following established practice in adaptive kernel density estimation for crowd counting^39^, which balances local density sensitivity with computational efficiency. The scaling factor 1/3 in Eq. (14) is selected based on cross-validation studies showing optimal performance in the range 0.2–0.4 for balancing heatmap smoothness and peak preservation^40,41^. To prevent extreme kernel sizes in sparse or ultra-dense regions, we constrain ${\sigma}_{i}$ within [2, 15] pixels, corresponding to approximately 0.1–0.75 m in real-world coordinates given our camera setup (Table 1).

This strategy concentrates kernels in dense areas to improve resolution and expands them moderately in sparse areas to stabilize visualization. As shown in Fig. 10, darker red in the heatmap indicates higher congestion, clearly identifying hotspots such as gate entrances and transfer channels—key indicators of potential risk zones.

By batch-processing historical video data over consecutive periods, the system constructs "density superposition maps" to analyze regional load trends during peak hours. These maps support subsequent spatial risk grading, point clustering, and serve as input features for training image-based crowd risk detection models.

Static density distribution alone is insufficient for identifying "potential risk points" and their dynamic evolution. High risk arises not from density alone but from "high density + rapid aggregation," which may trigger local surges or movement deflections, leading to congestion or stampedes. Thus, spatial aggregation features are extracted by fusing density estimation results with optical flow information from the PWC-Net trajectory model.

The PWC-Net model outputs pixel-level optical flow vectors, including motion direction and speed magnitude. For visualization and analysis, speed magnitudes are converted to heat values using pseudo-color encoding, where darker tones indicate faster movement (Fig. 11). Blue-green regions represent low-speed/static states, while orange-red regions denote rapid movement, enabling intuitive identification of potential aggregation points.

A "speed-density coupled aggregation feature map" is proposed to quantify risk points $R(X, t)$, which is weighted combines density values and speed magnitudes. Spatiotemporal distribution maps derived from this coupling reveal recurring peak-hour aggregation zones, typically near structural bottlenecks (narrow channels, escalator intersections, gates). These zones pose elevated safety risks due to delayed evacuation potential during emergencies. Additionally, "dynamic aggregation index sequences" are constructed by analyzing average speed trends over time, providing input for subsequent risk scoring models to quantify congestion or abnormal aggregation risks.

## Appendix 4

***Camera imaging model and ray-plane intersection calculation.*** All cameras must first be calibrated to obtain intrinsic and extrinsic parameters. Conventional cameras are modeled with a pinhole projection model, with intrinsic matrix $K$ (converting pixel coordinates to normalized direction vectors in the camera coordinate system) and lens distortion coefficients $d$. Spherical cameras are modeled using a polynomial equidistant projection, with the radial mapping relation given by:

15 $$\rho \left( \theta \right) = \sum\limits_{{k = 1}}^{m} {\alpha _{k} } {{\cdot}}\theta ^{k} , \in [0,\pi ),$$

where $\rho$ is the image-plane radius (distance from pixel to principal point), $\theta$ is the angle between the incident ray and optical axis, and ${\alpha}_{k}$ are polynomial coefficients. After distortion correction, each pixel $\mathbf{u}=(u, v)$ corresponds to a normalized direction vector $\mathbf{r}(\mathbf{u})$.

The extrinsics are represented by a rotation matrix ${R}_{i}$ and translation vector ${t}_{i}$. The optical center of the $i$-th camera in the world coordinate system is:

16 $$C_{i} {\text{ = }} - R_{i}^{{\mathrm{T}}} t_{i} ,$$

where ${C}_{i}$ denotes the position of the camera’s optical center in the world coordinate system. Accordingly, the ray equation formed by a pixel $\mathbf{u}$ is expressed as:

17 $${\mathrm{X(}}\lambda ){\text{ = }}C_{i} + \lambda R_{i} {\mathbf{r}}({\mathbf{u}}),$$

where λ is the ray length parameter, X(λ) represents the 3D point through which the ray passes, and $\mathbf{r}(\mathbf{u})={K}^{-1}\mathbf{u}$ denotes normalized direction vectors.

The station concourse floor can be approximated as a plane $\Pi$, whose equation is given by:

18 $$\Pi :n^{{\mathrm{T}}} X + c,$$

where, $n$ is the plane normal vector (typically a unit vector in the vertical direction), and $c$ denotes the plane constant term. The intersection point between the ray and the plane is calculated as:

19 $$\lambda ^{*} = \frac{{n^{{\mathrm{T}}} C_{i} + c}}{{n^{{\mathrm{T}}} R_{i} {\mathbf{r}}({\mathbf{u}})}},X^{*} = C_{i} + \lambda ^{*} R_{i} {\mathbf{r}}({\mathbf{u}}),$$

where, λ* is the scale factor of the intersection point, and ${X}^{*}$ stands for the 3D coordinates of the pixel $\mathbf{u}$ projected onto the floor. Through this geometric derivation, each pixel can thus be mapped to its corresponding point on the actual floor.

## Appendix 5

***Projection to Ground-Level Physical Quantities.*** In the pixel domain, the algorithm can generate a density heatmap ${D }_{i}^{pix}(\mathbf{u},t)$ and an optical flow field ${f }_{i}^{pix}(\mathbf{u},t)$. These need to be projected onto the ground domain.

1. Density Mapping.

The ground density is calculated using the following formula:

20 $$D_{i} (X,t) = \sum\limits_{{{\mathbf{u}} \in {\mathbf{N}}(X)}} {D_{i}^{{pix}} } ({\mathbf{u}},t){{\cdot}}\kappa ({\mathbf{u}} \to X),$$

where $\mathbf{N}(X)$ denotes the set of pixels falling within a ground grid cell $X$, and $K({\mathbf{u}} \to X)$ is an interpolation kernel function that ensures density conservation.

(2) Optical Flow to Velocity Conversion.

Let be the projection function $\mathbf{u}={\pi}_{i}(X, Y)$, and its Jacobian matrix is expressed as:

21 $$J_{i} (X,Y) = \frac{{\partial \pi _{i} }}{{\partial (X,Y)}}.$$

The relationship between pixel optical flow and ground velocity is given by:

22 $$\left[ {\begin{array}{*{20}c} {\Delta u} \\ {\Delta v} \\ \end{array} } \right] = J_{i} (X,Y)\left[ {\begin{array}{*{20}c} {\Delta X} \\ {\Delta Y} \\ \end{array} } \right].$$

The ground velocity is then derived as:

23 $$v_{i} (X,t) = \frac{1}{{\Delta t}}J_{i}^{{ - 1}} (X,Y)f_{i}^{{pix}} ({\mathbf{u}},t),$$

here, ∆_t_ is the time interval between consecutive frames, and ${v}_{i}(X, t)$ represents the velocity vector at the point in question.
